# The HIF-Mediated PI3K-AKT Signaling Pathway Is a Key Signaling Pathway Triggering Testicular Spermatogenic Disorders in Yaks with Cryptorchidism

**DOI:** 10.3390/vetsci13050468

**Published:** 2026-05-12

**Authors:** Dapeng Yang, Ligang Yuan, Haojun Sun, Jiman Wang, Yao Wang, Yubao Li

**Affiliations:** 1 Phage Research Center, College of Agriculture and Biology, Liaocheng University, Liaocheng 252000, China; dapengy9295@163.com (D.Y.); m15376985781@163.com (H.S.); 17860449921@163.com (J.W.); 17548889830@163.com (Y.W.); 2 College of Veterinary Medicine, Gansu Agricultural University, Lanzhou 730070, China; yuan2918@126.com

**Keywords:** yak, testis, cell adhesion molecules, tight connection, Sertoli cells, hypoxia-inducible factor (HIF)

## Abstract

Cryptorchidism is one of the major reproductive diseases affecting testicular function in yaks. However, the mechanisms underlying its impact on testicular spermatogenesis remain unclear. In this study, high-throughput transcriptomics (RNA-seq) and proteomics technologies were employed to analyze the key signaling pathways involved in cryptorchidism-induced spermatogenic dysfunction in yak, and a mouse model was established for validation. The results indicate that differentially expressed genes (DEGs) in the testes of yak with cryptorchidism are primarily enriched in the hypoxia-inducible factor (HIF) signaling pathway and the PI3K-AKT signaling pathway. Experimental results from the hypoxic mouse model indicate that the hypoxic environment remarkably raised HIF-1α content in the blood of mice while activating the PI3K-AKT signaling pathway, accompanied by decreased testicular expression of the cell adhesion molecules (CAMs) claudin 2, claudin 3, E-cadherin, and N-cadherin. Cell culture experiments showed that cell adhesion molecule expression was significantly downregulated when HIF-1α and PI3K expression were inhibited among mouse Sertoli cells, indicating that the HIF-1α/PI3K-AKT signaling pathway regulated cell adhesion molecule expression among mouse testes. Decreased CAMs directly affect tight junctions and the adhesion of spermatogenic and Sertoli cells, thus affecting sperm production and potentially also testis development. This study provides data to support research on the regulatory mechanisms involved in reproductive function and hypoxia adaptation in male animals in a low-oxygen environment.

## Simple Summary

Cryptorchidism is one of the main reproductive diseases of testicular spermatogenic dysfunction in yak. However, the specific mechanisms by which cryptorchidism affects spermatogenesis remain unclear. This study, through transcriptomic and proteomic analyses, identified the HIF-1α/PI3K-AKT pathway as the primary signaling pathway influencing spermatogenesis in cryptorchid testicles. By establishing a mouse hypoxia model, the study found that HIF-1α regulates cell adhesion molecule expression by testicular Sertoli cells through activation of the PI3K-AKT signaling pathway under hypoxic conditions, which in turn affects testicular spermatogenesis. This study provides data to support research on the regulatory mechanisms involved in reproductive function and hypoxia adaptation in male animals in a low-oxygen environment.

## 1. Introduction

Hypoxia-inducible factor-1α (HIF-1α) is one transcription factor that deals with hypoxic conditions and mediates cells’ adaptive reaction to hypoxia. HIF-1α expression and activity are regulated through several mechanisms, including the oxygen-sensitive hydroxylase system, post-transcriptional modification, and control of protein stability. Additionally, HIF-1α participates in other biological reactions, like cell adhesion. Impaired cell adhesion can result in marked pathological changes, including tumor proliferation, metastasis, and resistance to apoptosis [1,2]. Cell attachment to other cells or extracellular matrices is mediated by a multitude of proteins, notably CAMs, which act as intermediates for immobilizing cells to other surfaces. Adhesion molecules are broadly divided into five categories—integrins, selectins, cadherins, immunoglobulin superfamily (IgSF) members, and others, including mucins [3]. As Ca^2+^-dependent adhesion molecules, cadherins regulate cell-to-cell junction interactions [4]. Type I cadherins are classified into three major subtypes, including E-cadherin, N-cadherin, and P-cadherin. As essential functional molecules, they play a pivotal role in the formation and stabilization of intercellular junctions [5]. This biological process relies on the binding of cell surface molecules (e.g., integrins, selectins, and mucins) to adjacent cells or the basement membrane. Furthermore, such molecular interactions are indispensable for multiple physiological and pathological processes, such as cancer cell metastasis, tissue morphogenesis, and wound repair.

The protein kinase B (AKT)/phosphatidylinositol 3-kinase (PI3K) signaling pathway becomes a crucial regulatory player in several cell processes. PI3K is the initiator of the PI3K-AKT signaling pathway. Following stimulation by activated growth factors, encompassing epidermal growth factor, insulin, and insulin-like growth factor, PI3K is activated and changes phosphatidylinositol 4,5-bisphosphate (PIP2) to phosphatidylinositol 3,4,5-trisphosphate (PIP3) in cell membranes [6]. PIP3 acts as a second messenger, recruiting protein kinase B-dependent protein kinase 1 (PDK1) and mammalian target of rapamycin complex 2 (mTORC2) to the cell membrane, resulting in AKT activation [7,8]. Activated AKT can promote cell progress and inhibit apoptosis by phosphorylating substrates of regulatory proteins such as cyclins, apoptosis inhibitors from the Bcl-2 family, and mTOR, leading to their dissociation [9,10]. The HIF-1α/PI3K-AKT signaling pathway is a critical regulatory player in cell adaptation and response to hypoxic conditions. Some research has indicated that the PI3K-AKT signaling pathway regulates the activity and stability of HIF-1α through several mechanisms [11]. Moreover, HIF-1α acts as a key upstream molecule that initiates and activates the PI3K-AKT signaling cascade. It transmits biological signals through sequential phosphorylation of downstream pathway molecules, thereby coordinately regulating cell proliferation, apoptosis and metabolic reprogramming [12]. AKT boosts HIF-1α stabilization while improving its transcriptional activity by both phosphorylating it and inhibiting HIF-1α degradation pathways (proteasomal degradation, oxygen-dependent degradation) [13]. Cross-regulation between the PI3K-AKT and HIF-1α signaling pathways is critical to cellular adaptation and responses to hypoxic stress. Without adequate oxygen, the PI3K-AKT signaling pathway is activated, thereby enhancing the transcriptional activity of HIF-1α and regulating the expression of various genes related to cell survival, metabolic adaptation, and angiogenesis [14]. This signaling pathway also plays a critical part in diseases such as tumors and cardiovascular disorders [15,16].

Yak (*Bos grunniens*) is a unique livestock species in the Qinghai–Tibet Plateau of China. They can adapt to the severe cold and anoxic environment of the plateau, and are an indispensable economic resource for the residents of the Qinghai–Tibet Plateau [17,18]. Cryptorchidism is one of the main reproductive diseases of testicular spermatogenesis dysfunction in yak. The condition causes the testes to fail to descend properly into the scrotum, usually in the abdominal or inguinal canal, leaving the testes in a relatively hypoxic environment [19,20]. This abnormal physiological position not only affects the normal temperature regulation of the testes, but also leads to changes in the testicular microenvironment, which in turn triggers a series of complex biological responses [21]. This study focused on exploring the key signaling pathways of testicular spermatogenesis impairment in cryptorchid yaks. Through transcriptome analysis, we found that differentially expressed genes (DEGs) in the testes of cryptorchid yaks were mainly enriched in the hypoxia-inducible factor (HIF) signaling pathway and the PI3K-AKT signaling pathway, both of which are closely related to the environmental adaptation mechanism of organisms. As a key transcription factor under hypoxic conditions, HIF can respond to hypoxic stimulation and activate downstream signaling pathways, including the PI3K-AKT signaling pathway, thereby regulating cell proliferation, differentiation, apoptosis, and metabolism [22]. In cryptorchid yaks, the HIF-mediated PI3K-AKT signaling pathway may lead to spermatogenesis disorder by affecting the physiological function of cells in the testes, especially the activity of spermatogonia and Sertoli cells. The purpose of this study is to deeply analyze the specific mechanism of the HIF-mediated PI3K-AKT signaling pathway in the impairment of testicular spermatogenesis in cryptorchid yaks, and to provide new perspectives and data to support the study of reproductive function of male animals such as yaks and adaptation mechanisms in hypoxic environments.

## 2. Materials and Methods

The experimental protocol was approved by the Animal Ethics Committee of Gansu Agricultural University (Approval No. GSAU-Eth-VMC-2023-005). All sampling procedures are in compliance with the “Guidelines on Ethical Treatment of Experimental Animals” (2006) No. 398, set by the Ministry of Science and Technology, China.

### 2.1. Animals and Ethics

Yak testis samples were collected from a farm in Datong District, Qinghai Province, China, and informed consent was obtained from the farm owner for experimental use. A total of 20 adult male yaks (approximately 4 years old) were used in this experiment, including 10 yaks with cryptorchidism (all with unilateral cryptorchidism, left inguinal) in the cryptorchidism group and 10 normal yaks in the normal control group. Once the yaks were slaughtered, the testes were quickly removed, and the samples for transcriptomics testing were sent to Chengdu Nomi Metabolic Biotechnology Co. Adult male Kunming mice were supplied by Beijing Vital River Laboratory Animal Technology Co., Ltd. (Beijing, China). In total, 24 healthy male mice were equally and stochastically separated into two groups, namely, a control group, in which mice were cultivated within a standard environment, and a hypoxia group, in which the mice were fed in a hypoxia chamber (12% oxygen, 0.9% carbon dioxide, temperature 25 °C, humidity 55%, 12 h light cycle) for 14 days. Mice in the inhibitor-treated groups received concurrently either BAY87-2243 (No. HY-15836, MedChemExpress Biotechnology Inc., Monmouth Junction, NJ, USA) (1 mg∙kg^−1^∙day^−1^) or LY294002 (No. HY-10108, MedChemExpress Biotechnology Inc., Monmouth Junction, NJ, USA) (1.2 mg kg^−1^∙day^−1^) i.p. for 2 weeks [23,24]. The mice in the hypoxia group and the control group underwent blood collection of 0.1 mL every day for 2 weeks, and then, the mice were sacrificed, and the testicles were collected.

### 2.2. RNA Extraction and Transcriptomics

Testicular samples of yak were homogenized in 2 mL grinding tubes at 55 Hz for 30 s using a freezing grinder. The powder was lysed with 1 mL TRIzol reagent (G3013-100ML, Servicebio Biotechnology Co., Ltd., Wuhan, China), vortexed for 30 s, and combined with 1 mL chloroform for centrifugation (12,000 rpm, 15 min, 4 °C). The supernatant underwent chloroform re-extraction (0.2 mL), centrifugation, and isopropanol precipitation (0.5 mL of isopropanol was added to each 0.4 mL of supernatant). After washing and drying, RNA pellets were dissolved in RNase-free water (20–70 μL) and stored at −80 °C. The integrity of RNA was confirmed using either agarose gel electrophoresis or an Agilent 2100 Bioanalyzer (Fremont, CA, USA). Following polyA selection, mRNA was fragmented to 200–300 bp with magnesium ions. Double-stranded cDNA was generated from these fragments via random hexamer priming and amplified by PCR to a length of 300–400 bp. After quality assessment on an Agilent 2100 and fluorescence-based quantification, the libraries were subjected to paired-end sequencing on an Illumina HiSeq system. Differentially expressed genes (DEGs) were identified with thresholds of |log2FoldChange| > 1 and FDR < 0.05, followed by Gene Set Enrichment Analysis (GSEA) for functional annotation [25,26].

### 2.3. Enzyme-Linked Immunosorbent Assay (ELISA)

Blood was obtained from mice in both groups. After centrifugation, the supernatant was delivered to a centrifuge tube for testing. A mouse hypoxia-inducible factor ELISA kit was purchased from Wuhan Gilead Biotechnology Co., Ltd. (Wuhan, China) (NO. J24231).

### 2.4. Immunofluorescence and Immunohistochemistry

The mouse testicular tissue fixed in 4% paraformaldehyde solution was removed, rinsed under running tap water for 12 h, dehydrated via a graded ethanol series, embedded in paraffin, and sectioned. Paraffin sections were dewaxed with xylene, rehydrated using a descending ethanol gradient (100%, 90%, 80%, and 70% [*v*/*v*]), and subjected to antigen retrieval for 15 min. Sections were then incubated in 3% H_2_O_2_ solution at 37 °C for 15 min. Next, goat serum working solution was added dropwise, followed by incubation for 20 min at 37 °C. Then, the slices were incubated with rabbit polyclonal antibody (50 μL, 1:300 dilution) or 0.01 mol/L phosphate-buffered saline (PBS; negative control) overnight at 4 °C. Staining was carried out using a DAB staining kit for immunohistochemical experiments (G1212-200T, Servicebio Biotechnology Co., Ltd., Wuhan, China). The subsequent steps were performed in an environment protected from light. After rinsing with PBS, the secondary antibody (goat anti-rabbit IgG [H+L] Alexa Fluor 488) (ab150077; 1:1000; Abcam, Cambridge, UK) was added dropwise, followed by incubation for 1 h at 37 °C in the dark, and then rinsing with PBS. Subsequently, the same secondary antibody was again added dropwise, followed by incubation for 4 h at 37 °C, and rinsed five times with PBS for 5 min each cycle. Thereafter, the sections were incubated with the subsequent secondary antibody (goat anti-mouse IgG [H+L] Alexa Fluor 647; added dropwise) (ab150115; 1:1000; Abcam, UK) for 1 h at 37 °C, rinsed five times with PBS, 5 min per cycle, and finally incubated with DAPI (added dropwise) (ab104139; 1:800; Abcam, UK) for 1 h. After rinsing with PBS, the slides were sealed and observed directly under a laser-scanning confocal microscope [26] (Table 1).

**Table 1:** Immunofluorescence and WB primary antibody information.

Antibody Name	Manufacturer	Product Code	Dilution Ratio
HIF-1α	Affinity Biosciences, USA	BF8002	WB:1:400 IF/IH:1:500
HIF-1β	Affinity Biosciences, USA	DF6154	WB:1:400 IF/IH:1:500
PI3K	Affinity Biosciences, USA	AF6241	WB:1:400 IF/IH:1:500
AKT	Affinity Biosciences, USA	AF6261	WB:1:400 IF/IH:1:500
Claudin 2	Beijing Bioss Biotechnology Co., Ltd.	bs-23849R	WB:1:400 IF/IH:1:500
Claudin 3	Beijing Bioss Biotechnology Co., Ltd.	bs-2788R	WB:1:400 IF/IH:1:500
E-cadherin	Beijing Bioss Biotechnology Co., Ltd.	bs-10009R	WB:1:400 IF/IH:1:500
N-cadherin	Beijing Bioss Biotechnology Co., Ltd.	bsm-34125M	WB:1:400 IF/IH:1:500
SHBG	Abcam, Britain	ab126617	IF:1:500
SOX9	Abcam, Britain	ab185966	IF:1:500
β-actin	Beijing Bioss Biotechnology Co., Ltd.	bs-0061R	WB:1:800

### 2.5. Cell Culture and Treatment

The testicular tissue of normal mice was rinsed thrice in PBS consisting of 100 IU/mL penicillin and 100 mg/mL streptomycin. Then, Sertoli cells were segregated according to the method of Duan et al. [27]. Specifically, mouse testes were surgically removed, chopped into pieces, washed in DMEM/F12 culture medium, dissociated with collagenase (0.4 mg/mL) at 32 °C for 90–120 min, and rinsed by centrifugation (200 rpm, 10 min). Following 5 min precipitation, the cells were resuspended and rinsed thrice within DMEM/F12 culture medium (G4612-500ML, Servicebio Biotechnology Co., Ltd., Wuhan, China). After 5 min, the cell tubules were restored, with the cells resuspended thrice in DMEM/F12 medium. After treatment with 20 mL of medium containing 1 M glycine, 2 mM EDTA, and 20 IU/mL DNAse for 20 min at RT, contaminating mesenchymal cells were released; additionally, the cells were placed in PBS (pH 7.2) without Ca^2+^ or Mg^2+^ for 20 min, rinsed thrice through the DMEM/F12 culture medium, and cultivated within the DMEM/F12 culture medium containing collagen. After removing the supernatant, the precipitate in the tubule was treated with DNAse (0.05 mg/mL) and collagenase (0.4 mg/mL) for half an hour at 32 °C to segregate Sertoli cells. Although these testicular Sertoli cells purified using this process were not contaminated with Leydig or germ cells, 2% to 5% of the cells were peritubular muscle cells. After washing three times in DMEM/F12 medium, the Sertoli cells were collected and counted on one counter (Coulter Electronics, France). Subsequently, the cells were cultured in a 60 mm culture dish at 32 °C within DMEM/F12 culture medium supplemented with sodium bicarbonate (1.2 mg/mL), gentamicin (20 μg/mL), and 15 mM HEPES in a humid environment with 5% carbon dioxide. Insulin (2 μg/mL), transferrin (5 μg/mL), and tocopherol (10 μg/mL) were also integrated into the culture medium. Such a culture medium was altered every 48 h, during which time the nurtured support cells kept their specific activity.

Before tests, cells were cultivated in serum-free DMEM/F12 for 1 d, following which LY294002, a PI3K inhibitor, and BAY 87-2243, an HIF-1α inhibitor, were added to the corresponding 6-well plates.

### 2.6. Total RNA Extraction and qPCR

Total RNA was obtained from cells using a TRIzol reagent (ET111-01-V2, Beijing All-gold Biotechnology Co., Ltd., China) and instantly reverse-transcribed through a Prime Script RT kit. The amplified cDNA was subjected to qPCR using SYBR Green II PCR mix. Primers were devised and compounded by Shanghai Biotechnology Co., Ltd. Table 2 displays primer sequences. The relative fold change in the expression levels of the target genes was calculated using the 2^−ΔΔCt^ method (Ct: cycle threshold). The mRNA transcript abundance for each sample was measured three times [28].

### 2.7. Total Protein Extraction and Western Blot

In terms of the total protein extraction, cells were dissolved on ice for half an hour with high-efficiency RIPA buffer consisting of 1 mM phenylmethylsulfonyl fluoride (PMSF) and 10 μL/mL phosphatase inhibitor. After repeated shaking, the supernatant was collected following centrifugation at 8000 rpm for 15 min. Before western blot analysis, the collected protein was supplemented with the protein loading buffer and denatured in a metal bath for 20 min. Protein electrophoresis was performed after cooling.

### 2.8. Data Processing

The data was analyzed through SPSS 17.0 statistical software (IBM Technology, Chicago, IL, USA). A *t*-test was applied to intergroup comparisons. Histograms were plotted with GraphPad 9.0 software.

## 3. Results

### 3.1. DEG Enrichment Analysis

**Figure 1:**
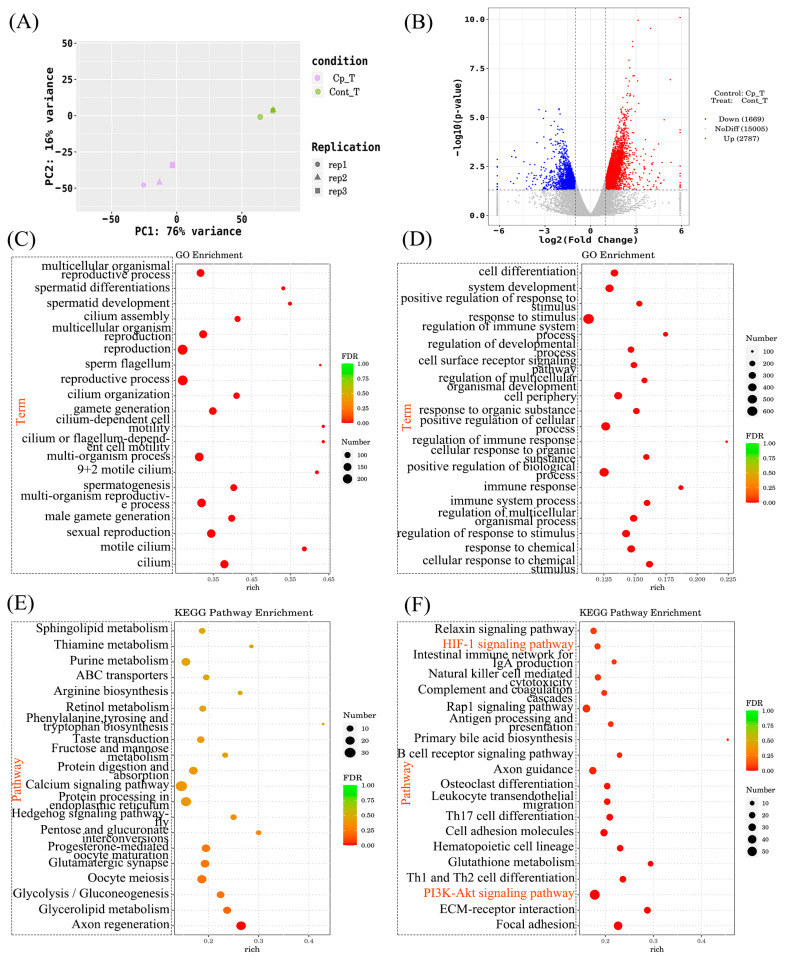
GO and KEGG enrichment analyses of the DEGs. (**A**) Principal component analysis graph of the samples. (**B**) Volcano plot depicting the DEGs. (**C**,**D**) Bubble charts illustrating the top 20 terms with the most significant upregulation (**C**) and downregulation (**D**) in the GO analysis. (**E**,**F**) Bubble charts presenting the top 20 pathways with the most significant upregulation (**E**) and downregulation (**F**) in the KEGG analysis.

Principal Component Analysis (PCA) results revealed no clustering between samples of the two groups (Figure 1A). According to the screening criteria of |log2FoldChange| > 1 and FDR < 0.05, a total of 4456 DEGs were detected in cryptorchid and normal testes, comprising 2787 upregulated genes and 1669 downregulated genes (Figure 1B, Supplementary Table S1). To evaluate the functions of differentially expressed genes (DEGs) in cryptorchid versus normal yak testes, the RNA-seq data were analyzed via gene ontology (GO), Kyoto Encyclopedia of Genes and Genomes (KEGG), and genome enrichment analysis (GSEA). GO term enrichment analysis revealed that 593 GO terms were significantly enriched (|log2FoldChange| > 1 and FDR < 0.05) across the molecular function (MF), biological process (BP), and cellular component (CC) categories. Within the BP category, the top enriched terms were sexual reproduction (upregulated) and cellular response to chemical stimuli (downregulated); within the CC category, the top enriched terms were cilia (upregulated) and cellular periphery (downregulated); and within the MF category, the top enriched terms were microtubule motility activity (upregulated) and binding (downregulated) (Figure 1C,D).

KEGG pathway enrichment analysis suggested that the DEGs were enriched in 248 pathways. The upregulated genes were mainly enriched in the cell cycle, calcium signaling pathway, glycerolipid metabolism, glycolysis/gluconeogenesis, axon regeneration, and other autophagy-related pathways. The downregulated genes were primarily enriched in the HIF-1 signaling pathway, the PI3K-AKT signaling pathway, CAMs, and Th1 and Th2 cell differentiation (Figure 1E,F).

### 3.2. Expression of Key Proteins in the PI3K-AKT/HIF-1α Signaling Pathway in Yak Testes

**Figure 2:**
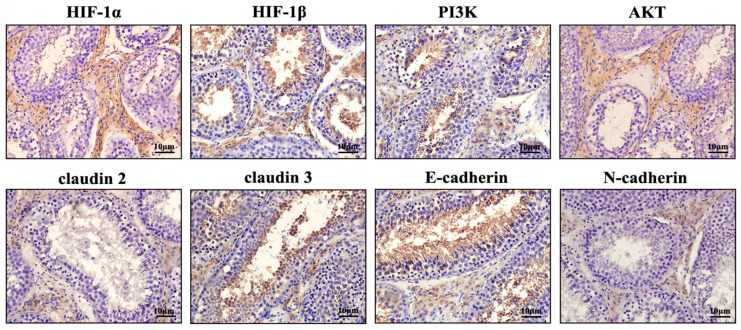
Expression and localization of key proteins in the PI3K-AKT/HIF-1α signaling pathway in normal yak testes.

According to the immunohistochemical analysis, it was shown that HIF-1α, HIF-1β, PI3K, AKT, claudin 2, claudin 3, E-cadherin, and N-cadherin were expressed in yak testes, with expression primarily detected in testicular interstitial and seminiferous tubules (Figure 2).

### 3.3. Histological Changes in Mouse Testes After Hypoxia Treatment

**Figure 3:**
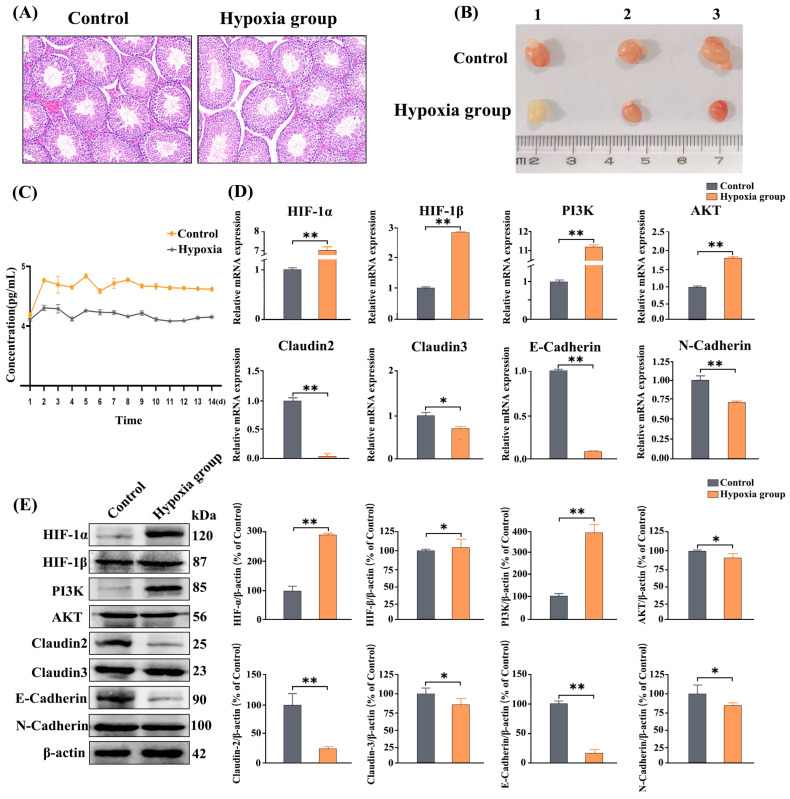
Effects of hypoxia on testicular development in mice. (**A**) Histological analysis of mouse testes before and after hypoxia treatment. (**B**) Testicular changes in mice after 14 days of hypoxia treatment. (**C**) Changes in HIF-1α contents in the blood of mice in the control and hypoxia groups. (**D**) The expression levels of genes and proteins in hypoxia-related pathways in the testes of mice in the control and hypoxia groups. (**E**) Gel map of proteins in hypoxia-related pathways in the testes of mice in the control and hypoxia groups. Data are presented as mean ± SEM. ** *p* < 0.01; * *p* < 0.05.

The histological staining uncovered that Leydig cells in the mouse testes decreased after hypoxia treatment. Although no remarkable difference emerged in seminiferous tubule diameter between hypoxia groups and controls, sperm in the lumen was significantly reduced in the latter (Figure 3A), as was the testicular volume (Figure 3B).

### 3.4. Determination of the HIF-1α Protein Content in the Blood of Mice

ELISA was applied for the sake of measuring the HIF-1α protein content in mouse blood. The results showed that the content of HIF-1α protein in the blood of mice significantly increased after one day in a hypoxic environment. Over the following 14 days, the total HIF-1α protein content was higher in the blood of mice in the hypoxia group than in that of mice in the control group. Moreover, although the HIF-1α protein content in the blood of mice in the hypoxia group showed a slight downward trend from day 8, it remained at a higher level compared with that in controls (Figure 3C). These findings demonstrate that hypoxia activates the HIF-1α signaling pathway in mice, resulting in a swift growth in HIF-1α content within the blood.

### 3.5. The Expression of Hypoxia Pathway-Associated Genes and Proteins

Quantitative real-time PCR analysis revealed that the relative mRNA levels of *HIF-1α*, *HIF-1β*, *PI3K*, and *Akt* were all significantly upregulated in the testes of mice following 14 days of hypoxia treatment, whereas the mRNA levels of the CAMs *claudin-2*, *claudin-3*, *E-cadherin*, and *N-cadherin* were significantly downregulated. Western blot analysis further showed that under hypoxic conditions, the protein expression levels of HIF-1α, HIF-1β, and PI3K were markedly upregulated, while those of Akt, claudin-2, claudin-3, E-cadherin, and N-cadherin were significantly downregulated (Figure 3D,E). These results indicate that, with the exception of Akt, the transcriptional levels of the examined genes are consistent with their translational levels, suggesting that Akt protein expression is subject to additional post-transcriptional regulation. Moreover, differences in mRNA stability and degradation rates may also contribute to the discrepancy between mRNA abundance and actual protein synthesis efficiency. Under hypoxic conditions, the upregulation of HIF-1α leads to decreased expression of key proteins in the PI3K-Akt pathway and CAMs, ultimately impairing tight junctions between testicular cells and thereby indirectly affecting spermatogenesis.

### 3.6. Immunofluorescence Analysis of Key Proteins in PI3K-AKT/HIF-1α Signaling Pathway in Mouse Testes

**Figure 4:**
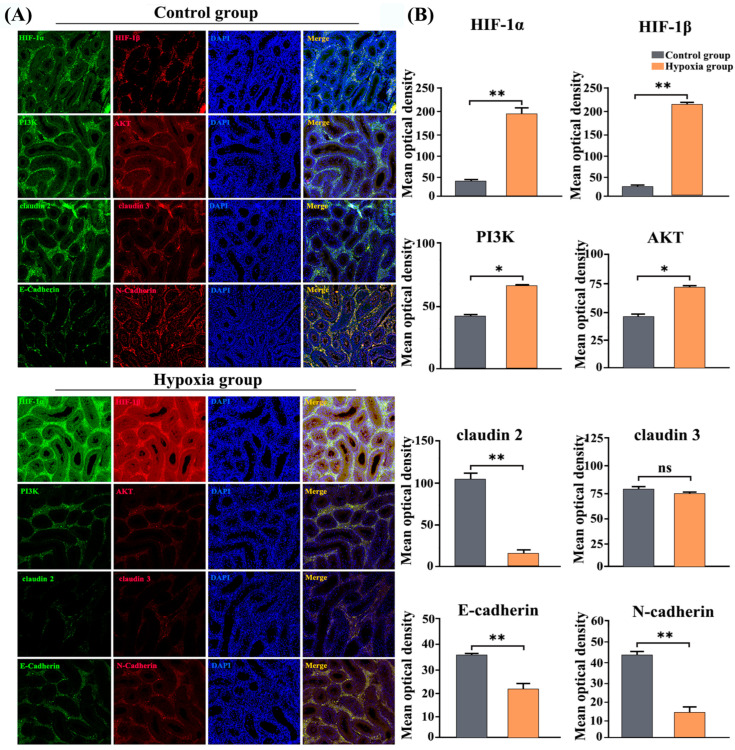
Analysis of the expression of key proteins in the PI3K-AKT/HIF-1α signaling pathway in mouse testes. (**A**) Immunofluorescence images of key proteins of PI3K-AKT/HIF-1α signaling pathway and CAMs in mouse testes after hypoxia treatment; (**B**) Bar chart of quantitative analysis of fluorescence intensity. Data are presented as mean ± SEM. ** *p* < 0.01; * *p* < 0.05; ns (not significant), *p* > 0.05.

Fluorescence intensity analysis showed that the expression of HIF-1α and HIF-1β in mouse testes was notably raised following hypoxia treatment, while cell adhesion molecule expression was significantly decreased (Figure 4A,B).

### 3.7. The Effect of Inhibition of Key Proteins in the PI3K-AKT/HIF-1α Signaling Pathway on the Expression of Testicular Adhesion Molecules

**Figure 5:**
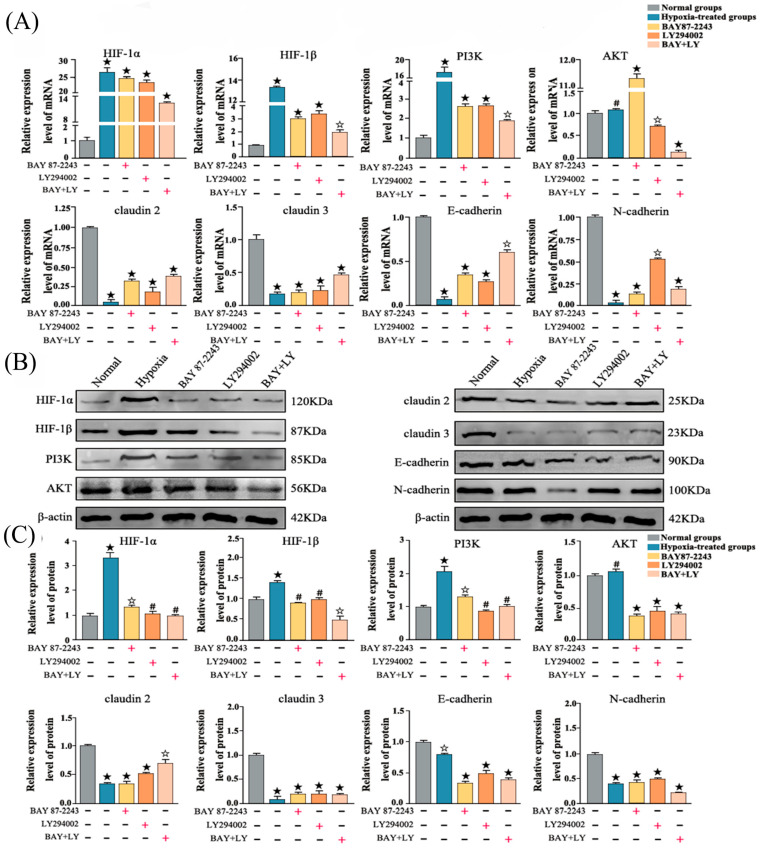
The effect of in vitro injection of inhibitors on the expression of adhesion molecules in mouse testicular cells. (**A**) The relative mRNA expression of key proteins in the PI3K-AKT/HIF-1α signaling pathway and CAMs after inhibitor treatment. (**B**) Western blot gels of key protein expression levels in the PI3K-AKT/HIF-1α signaling pathway and CAMs following inhibitor treatment. (**C**) After treatment with different inhibitors in mice, the protein expression levels of key molecules in the PI3K-AKT/HIF-1α signaling pathway and CAMs were plotted. Data are presented as mean ± SEM. ★, *p* < 0.01; ☆, *p* < 0.05; #, *p* > 0.05.

The expression of HIF-1α, HIF-1β and PI3K mRNA was significantly increased, but the expression of CAMs claudin 2, claudin 3, E-cadherin, and N-cadherin mRNA was significantly downregulated in the testes of hypoxia-treated mice. From the 7th day of hypoxia treatment, mice were injected with an appropriate amount of BAY 87-2243 and LY294002 for 14 days. Accordingly, it was indicated that the expression of HIF-1α, HIF-1β and PI3K mRNA in the testes of mice treated with hypoxia decreased after injection of inhibitors, but it still exceeded that in the healthy group, while the expression of the CAMs claudin 2, claudin 3, E-cadherin and N-cadherin mRNA was significantly upregulated (Figure 5A). The same is true for protein expression levels (Figure 5B,C). It is indicated that cell adhesion molecule expression in vivo closely correlates with the cell adhesion molecule/PI3K-AKT signaling pathway.

### 3.8. Culture and Identification of Mouse Sertoli Cells In Vitro

**Figure 6:**
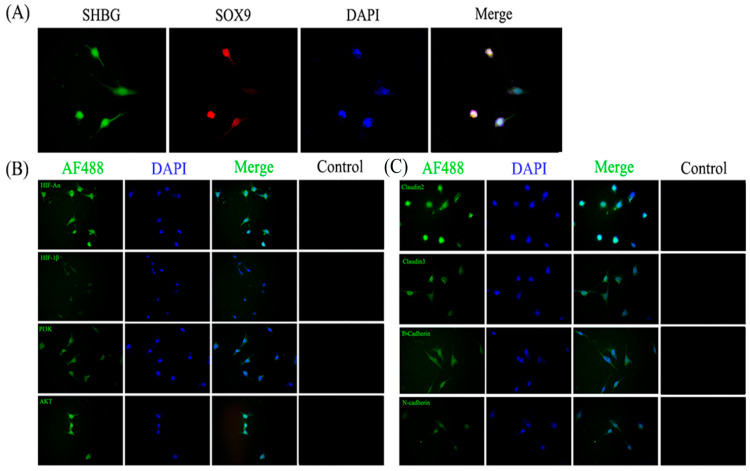
The expression of key proteins in the PI3K-AKT/HIF-1α signaling pathway and CAMs in mouse Sertoli cells. (**A**) Identification of mouse testicular Sertoli cells. (**B**) The expression of key proteins in the PI3K-AKT/HIF-1α signaling pathway in mouse Sertoli cells. (**C**) Expression of CAMs in mouse Sertoli cells.

To verify the purity of the isolated and cultured mouse testicular Sertoli cells, we performed an immunofluorescence analysis on Sertoli cells spread on coverslips using anti-SHBG (ab126617; 1:500; Abcam, UK) and anti-SOX9 (ab185966; 1:500; Abcam, UK) antibodies. We observed that Sertoli cells exhibited characteristic circular or square morphology, the cytoplasm showed green fluorescence associated with the anti-SHBG antibody, and the nucleus was stained red with the anti-SOX9 antibody; additionally, cell purity was high (Figure 6A).

### 3.9. The Expression of HIF-1α/PI3K-AKT Signaling Pathway-Associated Proteins Among Mouse Sertoli Cells

Immunofluorescence results showed that PI3K, AKT, HIF-1α, HIF-1β, claudin 2, claudin 3, E-cadherin, and N-cadherin proteins were expressed among mouse Sertoli cells and were localized to the cytoplasm (Figure 6B,C).

### 3.10. The Effect of the Inhibition of Key Proteins in the PI3K-AKT/HIF-1α Signaling Pathway on Sertoli Cell Adhesion

**Figure 7:**
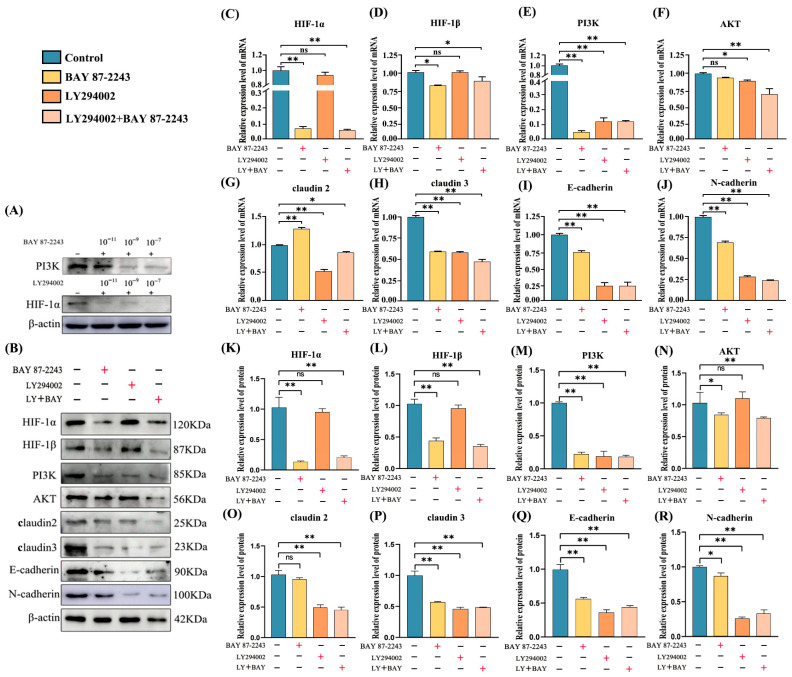
The effect of inhibiting the expression of key proteins in the PI3K-AKT/HIF-1α signaling pathway on the expression of adhesion molecules in mouse Sertoli cells. (**A**) Mouse Sertoli cell administration pattern diagram. (**B**) Determination of the optimal concentration of the two inhibitors BAY 87-2243 and LY294002. (**C**–**J**) The relative mRNA expression of key proteins in the PI3K-AKT/HIF-1α signaling pathway and CAMs after inhibitor treatment. (**K**) Western blot gels of key protein expression levels in the PI3K-AKT/HIF-1α signaling pathway and CAMs following inhibitor treatment. (**L**–**R**) After treatment of Sertoli cells with the different inhibitors, the protein expression levels of key molecules in the PI3K-AKT/HIF-1α signaling pathway and CAMs were plotted. Data are presented as mean ± SEM. ** *p* < 0.01; * *p* < 0.05; ns (not significant), *p* > 0.05.

BAY 87-2243 and LY294002, two protein inhibitors, were added separately to cultured mouse testis supporting cells (Figure 7A). To determine the optimal concentrations of protein inhibitors, different inhibitor concentrations (1 × 10^−11^, 1 × 10^−9^, and 1 × 10^−7^ nM/mL) were added to the medium. Consequently, it was shown that BAY 87-2243 and LY294002 exerted the most marked inhibitory effect at the concentration of 1 × 10^−7^ nM/mL (Figure 7B). Real-time quantitative PCR results showed that claudin 2 mRNA was upregulated 48 h after suppressing HIF-1α expression, while the comparative mRNA levels of PI3K, HIF-1β, claudin 3, E-cadherin, and N-cadherin were significantly downregulated (*p* < 0.01). After 48 h of PI3K protein inhibition, mRNA levels of HIF-1α and HIF-1β did not change significantly, but those of the other proteins were significantly downregulated (Figure 7C–J). Our western blot showed that suppressing HIF-1α protein expression did not affect the protein contents of claudin 2, whereas the relative protein expression levels of PI3K, AKT, HIF-1β, claudin 3, E-cadherin, and N-cadherin were significantly downregulated (*p* < 0.01). Furthermore, inhibiting the PI3K protein in mouse support cells for 48 h did not affect HIF-1α, HIF-1β, or AKT protein abundance, but significantly downregulated the expression of CAMs (Figure 7K–R). The above results show that a reduction in HIF-1α expression levels directly impacts the expression of pivotal proteins in the PI3K-AKT signaling pathway, thereby regulating the differential expression of mouse Sertoli-cell-adhesion-related molecules. Consequently, the diminished expression of adhesion molecules among Sertoli cells might be a potential contributing factor to impaired spermatogenesis or even the development of cryptorchidism.

## 4. Discussion

**Figure 8:**
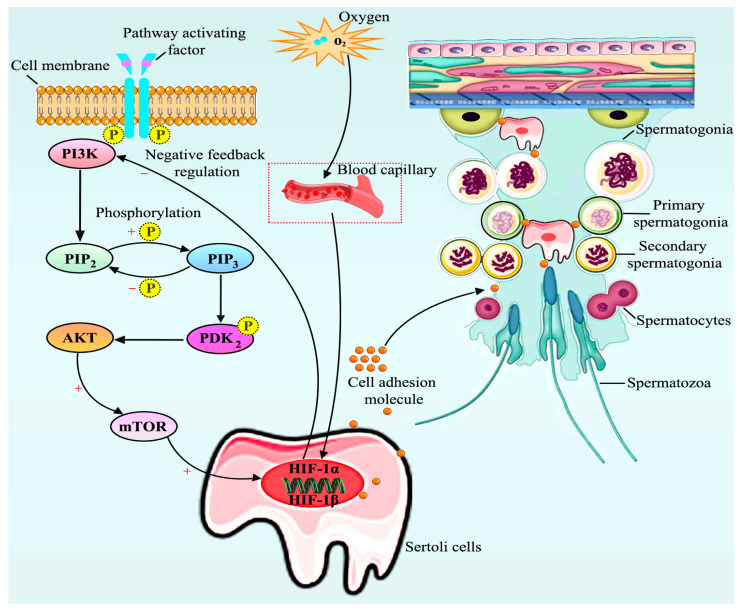
Schematic diagram showing how HIF-1α regulates the adhesion mechanism of mouse testicular supporting cells via the PI3K-AKT signaling pathway. Graphical abstract: Under hypoxia, blood oxygen content is decreased, which increases the protein concentration of HIF-1α; this, in turn, stimulates PI3K-AKT signaling pathway-mediated synthesis of Sertoli CAMs. Impaired synthesis of testicular adhesion molecules directly affects testicular spermatogenesis and can even induce a series of male reproductive system diseases, such as testicular atrophy or cryptorchidism.

Adhesion between cells is a necessary condition for multicellular organisms to organically connect numerous cells into a whole. Cell adhesion is a crucial biological process involving precise cell-to-cell and cell-to-matrix interactions. HIF-1α affects cell adhesion through various mechanisms, encompassing the modulation of cell adhesion molecule expression, alterations in extracellular matrix properties, the regulation of cell metastasis and adhesion, and the regulation of signaling pathways [29,30]. These effects highlight the important role of HIF-1α in both physiological and pathological processes. Research has shown that HIF-1α is capable of modulating cell-surface adhesion molecule expression. This is important for cell-to-cell and cell-to-matrix interactions. For example, in a diabetic-retinopathy-related study, it was found that HIF-1α can regulate CD18 expression on the surface of leukocytes as well as that of intercellular adhesion molecule-1 (ICAM-1) among vascular endothelial cells [31,32]. This indicates that HIF-1α participates in the modulation of cell adhesion. In this study, in vivo and in vitro data show that changes in HIF-1α expression directly affect cell adhesion molecule expression among mouse testicular Sertoli cells. The underlying mechanism might involve testicular hypoxia tolerance mechanisms regulated via the HIF-1α/PI3K-AKT signaling pathway (Figure 8).

Research has illuminated that the PI3K-AKT signaling pathway is capable of boosting spermatogenesis by enhancing the differentiation and progression of testicular Sertoli cells and spermatogonia [33,34,35], indicating that the PI3K-AKT signaling pathway is a key player in spermatogenic cell survival and growth. Known as protein kinase B, the AKT protein contributes to maintaining the function and number of spermatogenic cells by inhibiting apoptosis and promoting cell survival [36]. Additionally, the AKT signaling pathway engages in modulating hormone signals related to spermatogenesis. To be specific, follicle-stimulating hormone (FSH) and testosterone impact the maturation and role of spermatogenic cells through the PI3K-AKT pathway [37], while during spermatogenesis, this pathway also participates in the differentiation and migration of spermatogenic cells [38]. These processes are essential for normal sperm formation. Moreover, the PI3K-AKT pathway regulates the gene expression associated with spermatogenesis, affecting various phases of this process, including cell cycle regulation and morphogenesis [39]. Our research unveiled that the expression of four CAMs—claudin 2, claudin 3, E-cadherin, and N-cadherin—was significantly downregulated among mouse testicular supporting cells when PI3K protein expression was inhibited, suggesting that the aforementioned signaling pathway also indirectly modulates testicular spermatogenesis by affecting adhesion between testicular cells. In contrast, HIF-1α has an inhibitory effect during spermatogenesis, which was demonstrated by a decrease in the testicular spermatogenic cell apoptosis and the significant improvement in testicular spermatogenic function in VC rats following the silencing of the HIF-1α gene [40]. This suggests that HIF-1α might be a key player in spermatogenesis with regard to VC-induced male infertility. In agreement with this, we found that after 14 days of hypoxia treatment, HIF-1α expression was notably raised in mouse testes, while that of the CAMs claudin 2, claudin 3, E-cadherin, and N-cadherin was significantly downregulated. This downregulation in the levels of cellular adhesion molecules in the testes can directly affect the spermatogonial cells of the testes, thereby affecting testicular tight junctions and, consequently, testicular spermatogenesis.

As an important component of cellular hypoxic stress reactions, HIF-1α plays an indispensable part in sustaining tissue and cell oxygen equilibrium. In mammals, the stability of blood oxygen levels is critical for normal physiological functions. Research has illustrated that elevated expression of HIF-1α helps cells adapt to hypoxic environments. In this process, HIF-1α is modulated through the PI3K/AKT signaling pathway, which influences HIF-1α transcription [41,42]. Moreover, the synthesis of HIF-1α can also be upregulated when such a signaling pathway is activated, following which HIF-1α translocates to the nucleus while initiating VEGF transcription, thereby promoting neovascularization [43]. In comparison, our findings revealed that no variation occurred in the expression of HIF-1α/AKT and that the expression of PI3K was suppressed in mouse Sertoli cells, whereas the expression of AKT and PI3K was remarkably downregulated when HIF-1α expression was suppressed. This indicated that the AKT/PI3K signaling pathway was negatively regulated by HIF-1α.

CAMs are key players in diverse biological reactions, like cell growth, decomposition, and apoptosis. These molecules function through receptor–ligand binding, thus mediating cell-to-cell, cell-to-matrix and cell–matrix–cell adhesion. This adhesion is fundamental for cell identification, cell migration, signal transduction, cell progression, differentiation, and cell extension and motion [44,45]. In addition, CAMs form the molecular foundation of an array of pivotal pathogenetic and physiological reactions, like immunoreaction, coagulation, inflammation, and tumor transfer, as well as wound healing [46,47]. Claudin protein family members are key parts of close junctions and crucial players in cellular polarity, epithelial barrier function, cell motility, and cell-to-cell stability [48,49,50]. Research indicates that claudin 2 and claudin 3 are vital constituents of testicular cell tight junctions. Furthermore, Jhun et al. found that expression of claudin 2 and claudin 3 was prominently upregulated at protein and mRNA levels among induced canine cryptorchidisms [51]. Studies have shown that in the seminiferous epithelium, Sertoli cells form the blood–testis barrier (BTB) through specialized TJs, which are functionally coupled with adherens junctions (AJs) and desmosome-like junctions. CAMs such as N-cadherin, β-catenin, and junctional adhesion molecules (JAMs) are essential for the assembly and stability of these TJ–AJ complexes [52,53]. When CAM expression is downregulated—due to toxicants, hormonal disruption, or genetic manipulation—the structural integrity of the BTB is compromised. This leads to increased paracellular permeability and disruption of basal–adluminal compartmentalization [54]. Consequently, meiotic spermatocytes and post-meiotic spermatids, which depend on precise spatiotemporal support from Sertoli cells, lose their anchorage and detach prematurely into the tubular lumen [55]. Thus, the sequence is: CAM reduction → TJ opening (BTB injury) → germ cell detachment → apoptosis → hypospermatogenesis [56,57,58]. This direct pathway explains why even subtle changes in adhesion molecules can profoundly impair spermatogenesis without major testicular inflammation.

In this study, we found that claudin 2 and claudin 3 were expressed in cultured mouse testicular SCs, indicating that claudin 2 and claudin 3 were critical players in testicular spermatogenesis. Similarly, our discoveries illustrated that inhibiting PI3K, an upstream modulator of the HIF-1α/PI3K-AKT signaling pathway, caused a slight downregulation of claudin 2 and claudin 3 proteins, but their mRNA levels remained unchanged. However, when HIF-1α was inhibited, claudin 2 and claudin 3 protein expression showed a trend for downregulation, whereas their mRNA expression levels were highly significantly downregulated, indicating that claudin 2 and claudin 3 expression was regulated by the level of the intranuclear transcriptional activity of HIF-1α. Similarly, the expression of E-cadherin and N-cadherin is regulated by the transcriptional HIF-1α activity level in the nucleus. Indeed, E-cadherin and N-cadherin become the main markers of the epithelial–mesenchymal transition (EMT) and crucial CAMs [59,60,61]. The above-described signaling pathway disrupts tight junctions between both support cells and spermatogenic cells in the testes by negatively regulating CAMs in support cells. This disruption is an important reason for the impaired spermatogenesis in the testes under prolonged hypoxic conditions.

## 5. Conclusions

Differentially expressed genes in the testes of cryptorchid yaks were mainly enriched in the hypoxia-inducible factor (HIF) and PI3K-AKT signaling pathways, both of which are associated with environmental adaptation mechanisms. Hypoxia significantly increased HIF-1α levels in mouse blood, activated these pathways, and subsequently downregulated the expression of CAMs, including claudin-2, claudin-3, E-cadherin, and N-cadherin, in mouse testes. In vitro experiments further confirmed that inhibition of HIF-1α and PI3K reduced the expression of these adhesion molecules, indicating that their expression is regulated by the HIF-1α/PI3K-AKT signaling pathway. The decreased expression of CAMs disrupts tight junctions and adhesion between spermatogenic cells and Sertoli cells, thereby impairing spermatogenesis and testicular development.

## Figures and Tables

**Table 2:** Primer information list.

Gene Name	Sequences (5′ → 3′)	Product Length (bp)	Tm	Accession No.
PI3K	F:ACCATTACAAAGAAAGCCGGAC	180	58 °C	NM_174201.2
	R:GGGGCAGTGCTGGTGG			
Akt	F:GAGTCCTACCCCTGAATCTGC	198	59 °C	NW_005394100.1
	R:GCCAGGTTTTAATATATTCCCCTCG			
HIF-1α	F:GACAGAGCCGGCGTTTAGG	130	60 °C	NW_005393209.1
	R:CGACGTTCAGAACTCATCCTATT			
HIF-1β	F:CAGCCATCTTTCATTGGGATG	124	59 °C	DQ838047.1
	R:TGGTACCCCCTGACAGGAC			
Claudin 2	F:CTCCGTGAGTATCTGGTCGTTT	103	58 °C	NW_005396868.1
	R:CAGTGTCTCTGGCAAGCTGA			
Claudin 3	F:TGAGGTGGTTCTCCGAAACG	117	60 °C	NW_005393406.1
	R:GCAGCCTTCAGGTTCTCGAT			
E-cadherin	F:ATGTCCTGGGCAGAGTGAGA	127	60 °C	XM_005896515.2
	R:TGGAGCTTTAGATGCCGCTT			
N-cadherin	F:GGCCTTGCTTCAGGCGT	111	59 °C	NM_031333.2
	R:TGTCCTTCGTGCACATCCTT			
β-actin	F:GAGCGCAAGTACTCTGTGTG	159	59 °C	NM_007393.5
	R:GGTGTAAAACGCAGCTCAGTAA			

## Data Availability

The datasets used and analyzed during the current study are available from the corresponding author on reasonable request. All RNA-seq and proteomic data used in this study are available in the SRA database (ID: PRJNA1197394, https://www.ncbi.nlm.nih.gov/sra/PRJNA1197394, accessed on 10 March 2025).

## Supplementary Materials

The following supporting information can be downloaded at: https://www.mdpi.com/article/10.3390/vetsci13050468/s1, Table S1. Differentially expressed genes summary table.

## Abbreviations

The following abbreviations are used in this manuscript:

HIF-1α	Hypoxia-inducible factor
PI3K	Phosphatidylinositol 3-kinase
AKT	Protein kinase B
DEGs	Differentially expressed genes
CC	Cellular component
MF	Molecular function
BP	Biological process
GSEA	Gene Set Enrichment Analysis
GO	Gene ontology
KEGG	Kyoto Encyclopedia of Genes and Genomes
GSEA	Genome enrichment analysis
EMT	Epithelial–mesenchymal transition
